# Profile and factors associated with glycaemic control of patients with type 2 diabetes in Greece: results from the diabetes registry

**DOI:** 10.1186/s12902-020-0496-7

**Published:** 2020-01-28

**Authors:** Kyriakos Souliotis, Anastasios Koutsovasilis, Georgia Vatheia, Christina Golna, Sofia Nikolaidi, Erifili Hatziagelaki, Kalliopi Kotsa, Theocharis Koufakis, Andreas Melidonis, Athanasia Papazafiropoulou, Nikolaos Tentolouris, Evangelia Siami, Alexios Sotiropoulos

**Affiliations:** 1 Department of Social and Education Policy, University of Peloponnese, Corinth, Greece; 2 Health Policy Institute, 8, Agisilaou Str, 15123 Maroussi, Athens Greece; 3 3rd Department of Internal Medicine and Diabetes Center, General Hospital of Nikaia, Piraeus, Greece; 4 Innowth Ltd, Larnaca, Cyprus; 5 2nd Department of Internal Medicine-Propaedeutic, Research Institute and Diabetes Center “Attikon” University Hospital Medical School, National and Kapodistrian University of Athens, Athens, Greece; 6 Division of Endocrinology and Metabolism and Diabetes Center, 1st Department of Internal Medicine, Medical School, Aristotle University, AHEPA University Hospital, Thessaloniki, Greece; 7 1st Department of Internal Medicine and Diabetes Center, Tzaneio General Hospital of Piraeus, Athens, Greece; 8 1st Department of Propaedeutic Internal Medicine, Medical School, National and Kapodistrian University of Athens, Laiko General Hospital, Athens, Greece

**Keywords:** Type 2 diabetes mellitus, Glycaemic control, Registry, Profile

## Abstract

**Background:**

Strict glycaemic control early in the treatment process has been shown to reduce the occurrence of micro- and macro- vascular complications of diabetes in the long-term. Thus, treatment guidelines advise early intensification of treatment to achieve glycaemic control goals. However, evidence in Greece suggests that, despite guideline recommendations, glycaemic control among patients with T2DM remains challenging. This study presents the demographic and clinical characteristics of patients with T2DM in Greece using data from an electronic registry designed specifically for this treatment category and investigates the factors that are independently associated with glycaemic control.

**Methods:**

This is a multi-center, observational, cross-sectional study to investigate epidemiological and clinical factors affecting glycaemic control among patients with T2DM in Greece. Data was collected via a web-based disease registry, the Diabetes Registry, which operated from January 1st to December 31st, 2017. Five large specialized diabetes centers operating in Greek hospitals participated in the study.

**Results:**

Data for 1141 patients were retrieved (aged 63.02 ± 12.65 years, 56.9% male). Glycaemic control (Hb1Ac < 7%) was not achieved in 57.1% of patients. Factors independently associated with poor glycaemic control were: family history of diabetes [OR: 1.53, 95% CI: 1.06–2.23], BMI score between 25 to 30 [OR: 2.08, 95% CI: 1.05–4.13] or over 30 [OR: 2.12, 95% CI 1.12–4.07], elevated LDL levels [OR: 1.53, 95% 1.06–2.21] and low HDL levels [OR: 2.12, 95% CI: 1.44–3.12]. Lastly, use of injectable antidiabetic agents (in monotherapy or in combination) was less likely to be associated with poor glycaemic control versus treatment with combination of oral and injectable agents [OR: 0.50, 95% CI: 0.24–1.01]. This association was found to be marginally statistically significant.

**Conclusion:**

Inadequate lipid control, family history of diabetes and presence of obesity (ΒΜΙ ≥ 30 kg/m^2^) were associated with poor glycaemic control among study sample, whereas use of injectable antidiabetic agents was less likely to be associated with poor glycaemic control. These findings indicate how complex optimal glycaemic control is, highlighting the need for tailored interventions in high-risk subpopulations with T2DM.

## Background

Diabetes Mellitus (DM), a metabolic disorder primarily characterized by hyperglycemia, has constituted one of the most critical challenges to health systems all over the world [1–3]. According to the 2017 IDF Diabetes Atlas, the global prevalence of DM was estimated at 8.4% in adults aged 18–99 years. It is also a major contributor to global mortality with 5 million deaths attributed to complications related to diabetes during that year [4]. Type 2 Diabetes Mellitus (T2DM) accounts for 90–95% of all cases of diabetes and is, therefore, the most common type of DM [5, 6]. Risk factors include genetic predisposition [7–9] and lifestyle factors, mainly obesity, [10] physical inactivity [11] and smoking [12, 13]. The global prevalence of T2DM is expected to increase in both developed and developing countries over the next decades [14, 15].

In Greece, the prevalence of T2DM has been the subject of various epidemiological studies. According to recent estimations based on real-world data, 694,357 patients received prescribed medication for T2DM in 2015, accounting for 6.8% of the country’s population [16]. Furthermore, according to another study in rural, urban and suburban Greek populations, T2DM was associated with age over 40 years, obesity (BMI ≥ 30), personal history of smoking in the past and low socioeconomic status [17]. The economic burden of the disease is equally high. A 2014 study estimated the annual cost of each patient with diabetes in Greece at €7111. The cost was significantly higher in patients with poor glycaemic control (hemoglobin A1c [HbA1c] > 7%). The largest contributor to disease cost were complications of diabetes and comorbidities [18].

Strict glycaemic control early in the treatment process has been shown to reduce the occurrence of micro- and macro- vascular complications of diabetes in the long-term [19–22]. Thus, treatment guidelines advise early intensification of treatment to achieve glycaemic control goals, as individualized for each patient [23]. However, evidence in Greece suggests that, despite guideline recommendations, glycaemic control among patients with T2DM remains challenging in both the trial setting and the real world [24, 25]. This was evident in a 2013 Greek study among 6631 randomly selected patients with T2DM of whom 59% were found not to have achieved the target goal of HbA1c < 7%. More specifically, 44.7% of patients had an HbA1c level of 7–7.9 and 14.3% had an HbA1c > 8% [26]. Equally, another 2015 study showed that adequate glycaemic control had not been achieved in 32.9% of patients treated in 25 primary care sites [27], whilst a later study (2017) confirmed that 42% of study patients had not achieved the HbA1c target of < 7% [28].

A disease registry integrates a variety of information such as demographic characteristics, laboratory test results, clinical data, comorbidities and follow-up information to gather valuable information on the trends and management of chronic diseases [29]. Diabetes registries have been used as a tool to assess the epidemiologic profile of patients with diabetes and the quality of specialist care provided in various medical centers [30].

In the present study, five major diabetes units operating in large, public hospitals enrolled patients in a web-based disease registry, designed and developed to prospectively monitor and report on key disease indicators. The main objective was to explore current epidemiologic trends of the disease in Greece. Data regarding patient characteristics, co-morbidities and glycaemic control of participants were also collected. In addition, the study explored factors affecting the degree of glycaemic control among patient population to further recognize patient groups in need of intensive monitoring.

## Methods

### Sample and setting

This was a multi-center, cross-sectional observational study conducted in Greece. Participating centers were chosen as follows: out of 13 operating specialized diabetes centers in Attica, Piraeus, Macedonia and Thrace regions [31], six were contacted based on their special interest in diabetes research and their large geographic population coverage. Five out of six participated in the study. Study sample was comprised of patients with T2DM, both treatment naïve and treatment experienced, receiving diabetes treatment in these five participating centers.

All outpatient subjects, who were older than 18 years, diagnosed with T2DM and scheduled for a routine office visit during the time period between January 2017 and December 2017, were eligible to participate. Diagnosis of T2DM was based on the criteria proposed by World Health Organization (WHO) [32]. Patients were recruited on their first consultation in the study period (index visit). Additional data were also collected during follow up consultations, if patients visited the centers more than once during the study period.

### Ethics

All eligible patients signed informed consent forms prior to enrolment. The study was approved by the Research Committee board of the University of Peloponnese.

### Research tool

All data was collected with the use of a web-based diabetes registry that was specifically designed and developed to support this study. The Diabetes Registry was developed through a collaboration of the University of Peloponnese and the Medical School of the University of Athens. The research tool used was developed on the basis of international best practice, published, disease risk indices [33, 34] as validated by expert clinicians on the field. The registry database was maintained on a dedicated, secure, fully encrypted server in the University of Peloponnese. All and any data exchanges were fully encrypted. Access was provided to one researcher, a medical expert, in each participating diabetes center. A separate electronic medical file was created for each eligible patient on day 0. At every follow up visit, the patient file was re-accessed and follow-up information was uploaded. This information included recent lab results, changes in pharmacological treatment and any new events or hospitalizations. This analysis reports on information recorded during the first patient visit only.

The registry recorded:

- *Demographic characteristics****:*** Gender, age, educational level, place of residence, family and employment status.
- *Clinical characteristics:* Duration and family history of T2DM (a patient was defined as having family history of diabetes if one or both of his/her parents or/and any of his/her siblings were diagnosed with T2DM, at any time in the past), treatment modalities, presence of established main comorbidities (macro-vascular disorders), severe risk factors for Cardiovascular disorders (CVD) (hypertension, dyslipidemia or both), metabolic disorders (hypothyroidism and hyperthyroidism) and diabetes related complications (retinopathy, diabetic foot, erectile dysfunction and peripheral neuropathy). Presence of concomitant diseases was self-reported. Data regarding Body Mass Index (BMI) and waist circumference were also recorded. Patients were categorized based on their BMI score as follows: participants with BMI score less than 25 kg/m^2^ were defined as normal and with BMI score between 25 kg/m^2^ and 30 kg/m^2^ as overweight. Patients with obesity were defined as having BMI score equal/over 30 kg/m^2^. Waist circumference target was set at 80 cm for women and 90 cm for men.
- *Laboratory test results:* Levels of HbA1c, blood pressure (BP), high-density lipoprotein (HDL), low density lipoprotein (LDL), triglycerides (TG) and estimated Glomerular Filtration Rate (e-GFR) were assessed. EPI equation was used for the calculation of e-GFR. Glycaemic control was defined as adequate if HbA1c was less than 7%. Only patients who had a recent (during the last 12 months) laboratory test for HbA1c were included. For BP control target was set at less than 130/80 mm/Hg. Regarding lipid control, HDL levels of more than 40 mg/dl, LDL levels of less than 100 mg/dl and TG levels of less than 150 mg/dl were set as optimal. Participants were classified as having dyslipidemia if their serum lipids levels were other than optimal as described above and/or if they were on treatment with lipid lowering medications. Moreover, participants were classified as suffering from hypertension if they reported a previous diagnosis of hypertension or/and were receiving anti-hypertensive medication.
- *Lifestyle behaviors:* Alcohol and tobacco consumption, physical activity and dietary habits were recorded. The dietary and physical activity factors were selected from the Diabetes Prevention toolkit [33, 34]. Item selection was based on their clinical importance for the pertinent disease category, as defined by the clinical experts who collaborated in the development of the registry

The diabetes registry was piloted to 100 patients to assess its validity. Pilot study results were evaluated by a group of experts. Patients who participated in the pilot study were excluded from the final analysis.

### Statistical analysis

A descriptive and inferential analysis was performed on study data. Absolute and relative frequencies were used to describe categorical variables. Continuous variables were expressed using mean ± SD if normally distributed. Kolmogorov Smirnov criterion was used to test the normality of distributions.

All socio-demographic and clinical characteristics, laboratory measurements and lifestyle behaviors were univariately associated with glycaemic control (a cut-off point of HbA1c ≥ 7% was used). Patients diagnosed 6 or less months ago were excluded from further analyses. Chi-square tests were performed to test the association amongst categorical variables and independent t-test criterion was used to test for the association between a continuous and a categorical variable. All variables that yield statistically significance at the 5% level were entered into a multiple logistic regression model. Age, sex, BMI and duration of T2DM were entered into the final model as these are factors of pertinent clinical importance to this disease area and were considered to act as confounders. Adjusted Odds Ratios (OR) along with 95% Confidence Intervals and *p*-values are presented. All statistical analyses were conducted using SPSS v. 25 (Armonk, NY: IBM Corp.).

## Results

### Demographic and clinical characteristics

Study population consisted of 1141 patients with T2DM. Mean age was 63.02 ± 12.65 years. Most of the patients were male (56.9%), living in urban areas (95.8%), had completed mandatory education (77.5%) and were economically inactive (i.e. pensioners or students) at the time of the study (63.5%). 81.1% were married and almost half (45.4%) of them had children. Participants’ socio-demographic characteristics are presented in Table 1.

**Table 1:** Demographic characteristics

Demographic characteristics	
Gender (N, %)	
Men	649 (56.9)
Women	492 (43.1)
Urbanicity (N, %)	
Rural	44 (4.3)
Urban	970 (95.7)
Age (Mean ± SD; years)	63.02 ± 12.65
Educational level (N, %)	
Low (≤12 years of education)	410 (77.5)
High (> 12 years, college or university)	119 (22.5)
Marital status (N, %)	
Single	117 (17.4)
Married	546 (81.1)
Divorced/Widowed	10 (1.5)
Employment status (N, %)	
Employed	154 (22.6)
Economically inactive/ Student	433 (63.5)
Unemployed	95 (13.9)

Most patients had a T2DM diagnosis for more than 10 years (40.7%) and were categorized as obese based on their Body Mass Index score (BMI > 30; 61.8%). The majority of patients were on oral antidiabetic agents only (67.7%) and used 1 antidiabetic agent (66.3%). Family history of diabetes was reported by almost 1 in 2 participants (48.6%).

61.5 and 58.9% out of total study sample reported previous diagnosis of dyslipidemia and hypertension, respectively. Moreover, 43.6% of sample reported suffering from both of these conditions. 247 (21.6%) patients had a personal history of coronary artery disease, defined as stable or unstable angina, NSTEMI or STEMI. 94 (8.2%) had been diagnosed with heart failure. 53 (4.6%) had suffered from an ischemic or hemorrhagic stroke and another 50 (4.4%) from a transient ischemic attack (TIA). Hypothyroidism was present in 182 (16%) of the patients, while hyperthyroidism in 21 (1.8%). Sample’s clinical characteristics are presented in detail in Table 2.

**Table 2:** Clinical Characteristics and presence of comorbidities

Clinical characteristics	
Family history of diabetes (N, %; Yes)	**555 (48.6)**
T2DM duration	
0–6 months	109 (9.6)
6 months to 5 years	351 (30.8)
6–10 years	217 (19.0)
≥ 10 years	464 (40.7)
Dyslipidemia (N, %; Yes)	702 (61.5)
Hypertension (N, %; Yes)	682 (59.8)
Treatment modality (N, %)	
Diet and exercise	58 (5.1)
Oral antidiabetic agents only	773 (67.7)
Injectable antidiabetic agents only	131 (11.5)
Combination of oral and injectable antidiabetic agents	180 (15.8)
No. of antidiabetic agents used (N, %)	
1	719 (66.3)
2	279 (25.7)
≥ 3	86 (7.9)
Hyperthyroid (N, %; Yes)	21 (1.8)
Hypothyroid (N, %; Yes)	182 (16.0)
Coronary artery disease (N, %; Yes)	247 (21.6)
Heart failure (N, %; Yes)	94 (8.2)
Atrial fibrillation (N, %; Yes)	94 (8.2)
Stroke (N, %; Yes)	53 (4.6)
Transient ischemic attach (N, %; Yes)	50 (4.4)
Peripheral arterial disease (N, %; Yes)	73 (6.4)
Peripheral neuropathy (N, %; Yes)	74 (6.5)
Erectile dysfunction (N, %; Yes)	6 (0.5)
Diabetic foot (N, %; Yes)	25 (2.2)
Retinopathy (N, %; Yes)	57 (5)
eGFR^a^ (Mean ± SD; ml/min/1.73m^2^)	67.70 ± 17.05

*CKD* chronic kidney disease, *eGFR* estimated glomerular filtration rate, *T2DM* type 2 diabetes mellitus

^a^Estimated using CKD-EPI equation

### Lifestyle behaviors

Almost 1 in 3 patients were smoking and 1 in 5 had ceased smoking during the last 12 months (20.5%). Only 14.7% of total sample had been engaging in a physical activity for at least 30 min per day or 3 times per week. The vast majority of participants did not follow a nutritious diet, as only 10.9 and 13.5% of responders reported consuming 3 or more servings of whole grain cereals daily and vegetable oil most of the days, respectively. Almost one in two (47.8%) reported daily consumption of 3 servings of processed starch. Only 9.7% of study participants reported drinking one or more alcoholic drinks per day, with the majority consuming zero alcohol on a daily basis (Table 3).

**Table 3:** Sample’s lifestyle behaviors

Lifestyle behaviors	
BMI (N, %; kg/m^2^)	
< 25.0	106 (9.9)
25.0–29.9	303 (28.3)
≥ 30	663 (61.8)
Smoking (N, %)	
No	444 (47.8)
Former	190 (20.5)
Yes	294 (31.7)
Physical activity^a^	
No	973 (85.3)
Yes	168 (14.7)
Alcohol consumption per day (N,%)	
0 drinks	1030 (90.3)
> 1 drink	111 (9.7)
Processed starch, 3 servings per day (N, %)	
No	596 (52.2)
Yes	545 (47.8)
Whole grain cereals, 3 servings per day (N, %)	
No	1017 (89.1)
Yes	124 (10.9)
Consuming or cooking with vegetable oil, most days (N, %)	
No	987 (86.5)
Yes	154 (13.5)

*BMI* body mass index

^**a**^Moderate to rigorous physical activity for at least 30 min daily, at least 3 times/week

### Glycaemic control

Poor glycaemic control was assessed using an HbA1c threshold of 7%. One hundred nine patients with less than 6 months with a T2DM diagnosis were excluded from this analysis so as not to influence the results. Seven hundred fifty-five out of the total sample had a recent (during the last 12 months) HbA1c measurement. Only 42.9% of patients assessed achieved HbA1c levels of less than 7%.

Univariate analyses showed that poor glycaemic control was associated with family history of diabetes (*p* = 0.045) and the type of pharmacological treatment (*p* = 0.010). In addition, the following laboratory measurements were found to be associated with glycaemic control: LDL (*p* = 0.047), HDL (*p* < 0.001) and TG (p < 0.001) (Table 4).

**Table 4:** Socio-demographic and clinical factors associated with glycaemic control

	HbA1 < 7 (*N* = 294)	HbA1 ≥ 7 (*N* = 392)	*p*-value
*Socio-demographic characteristics*			
Age (Mean, SD; years)	62.99 (12.71)	63.24 (12.37)	.788
Sex (N, %)			
Male	182 (45.7)	216 (54.3)	.074
Female	112 (38.9)	176 (61.1)	
Urbanicity (N, %)			
Rural	9 (33.3)	18 (66.7)	.316
Urban	250 (43.1)	330 (56.9)	
Marital Status (N, %)			
Married	124 (39.2)	192 (60.8)	.639
Divorced	3 (50.0)	3 (50.0)	
Single	34 (44.4)	40 (55.6)	
Educational Level (N, %)			
≤ 12 years of education	100 (41.7)	140 (58.3)	.643
> 12 years of education (college or university)	30 (38.6)	43 (61.4)	
Employment status (N, %)			
Employed	34 (37.0)	58 (63.0)	.648
Economically inactive/student	110 (42.1)	151 (57.9)	
Unemployed	25 (43.1)	33 (56.9)	
*Clinical characteristics*			
T2DM duration (N, %)			
6 months to 5 years	97 (40.9)	140 (59.1)	.677
6–10 years	63 (42.3)	86 (57.7)	
≥ 10 years	134 (44.7)	166 (55.3)	
Family history of diabetes (N, %)			
No	166 (46.5)	191 (53.5)	**.045**
Yes	128 (38.9)	201 (61.1)	
Dyslipidemia (N, %)			
No	115 (45.1)	140 (54.9)	.380
Yes	179 (41.5)	252 (58.5)	
Hypertension (N, %)			
No	113 (42.0)	156 (58.0)	.752
Yes	181 (43.4)	236 (56.6)	
Pharmacological treatment (N, %)			
Oral antidiabetic agents only	187 (40.6)	274 (59.4)	**.010**
Injectable antidiabetic agents only	47 (58.0)	34 (42.0)	
Oral and injectable antidiabetic agents	44 (38.9)	69 (61.1)	
No. of antidiabetic agents (N, %)			
1	177 (43.3)	232 (56.7)	.843
2	79 (41.4)	112 (58.6)	
≥ 3	22 (40.0)	33 (60.0)	
BP (N, %; (mm/Hg)			
< 130/80	82 (42.1)	113 (57.9)	.892
≥ 130/80	116 (41.4)	164 (58.6)	
Body mass index (N, %; kg/m^2^)			
< 25.0	32 (48.5)	34 (51.5)	.420
25.0–29.9	80 (44.7)	99 (55.3)	
≥30	161 (40.9)	223 (59.1)	
LDL (N, %; mg/dl))			
< 100	143 (47.5)	158 (52.5)	**.047**
≥ 100	114 (39.3)	176 (60.7)	
HDL (N, %; mg/dl)			
> 40	191 (50.4)	188 (49.6)	**<.001**
≤ 40	74 (32.3)	155 (67.7)	
TG (N, %; mg/dl)			
< 150	184(53.2)	162 (46.8)	**<.001**
≥ 150	85 (31.3)	187 (68.8)	
Abdominal circumference (N, %; cm)			
Target not achieved	127 (41.1)	182 (58.6)	.725
Target achieved	71 (42.8)	95 (57.2)	
*Lifestyle behaviors*			
Smoking (N, %)			
No	117 (42.7)	157 (57.3)	.953
Former	44 (41.5)	62 (58.5)	
Yes	69 (41.3)	98 (58.7)	
Physical activity^‡^			
No	256 (42.9)	341 (57.1)	.974
Yes	38 (42.7)	51 (57.3)	
Alcohol consumption per day (N, %)			
0 drinks	271 (43.6)	351 (56.4)	.289
> 1 drink	23 (35.9)	41 (64.1)	
Processed starch, 3 servings per day (N, %)			
No	156 (44.1)	198 (55.9)	.508
Yes	158 (41.6)	194 (58.4)	
Whole grain cereals, 3 servings per day (N, %)			
No	259 (42.4)	352 (57.6)	.480
Yes	35 (46.7)	40 (53.3)	
Consuming or cooking with vegetable oil, most days (N, %)			
No	252 (42.9)	335 (57.1)	.925
Yes	42 (42.4)	57 (57.6)	

In bold if statistically significant at the *p* < 0.05 or *p* < 0.001 level

*BP* blood pressure, *LDL* low-density lipoprotein, *HDL* high-density lipoprotein, *TG* triglycerides

‡ Moderate to rigorous physical activity for at least 30 min daily, at least 3 times/week

Factors independently associated with poor glycaemic control in our sample are depicted in Table 5. After adjusting for all other variables in the model, the following factors were found to be independently associated with poor glycaemic control: family history of diabetes [OR: 1.53, 95% CI: 1.06–2.23], BMI score between 25 to 30 kg/m^2^ [OR: 2.08, 95% CI: 1.05–4.13] or over 30 kg/m^2^ [OR: 2.14, 95% CI 1.12–4.07], elevated LDL levels [OR: 1.53, 95% 1.06–2.21] and low HDL levels [OR: 2.12, 95% CI: 1.44–3.12]. Lastly, use of injectable antidiabetic agents was less likely to be associated with poor glycaemic control versus the reference category [OR: 0.50, 95% CI: 0.24–1.01]. The former association was found to be marginally statistically significant.

**Table 5:** Multiple logistic Regression: Factors influencing glycaemic control (*N* = 591)

	OR	95% CI	*p*-value
Family history of diabetes			
No	Ref.		
Yes	**1.53**	**1.06–2.23**	**.025**
Duration of T2DM			
1–5 years	Ref.		
5–10 years	1.10	.66–1.81	.717
≥ 10 years	.92	.59–1.43	.721
BMI score			
BMI < 25 (kg/m^2^)	Ref		
BMI 25–30	**2.08**	**1.05–4.13**	**.035**
BMI > 30	**2.14**	**1.12–4.07**	**.021**
Gender			
Men	Ref.		
Women	1.16	.80–1.69	.425
Age	1.00	.99–1.02	.607
HDL levels (mg/dl)			
HDL > 40	Ref.		
HDL ≤ 40	**2.12**	**1.44–3.12**	**< 0.001**
LDL levels (mg/dl)			
LDL < 100	Ref.		
LDL ≥ 100	**1.53**	**1.06–2.21**	**.024**
Pharmacological treatment			
Oral antidiabetic agents	.85	.51–1.41	.528
Injectable antidiabetic agents	**.50**	**.24–1.01**	**.054**
Combination of oral and injectable antidiabetic agents	Ref.		

In bold if statistically significant at the *p* < 0.05 or *p* < 0.001 level

*BMI* body mass index, *HDL* high-density lipoprotein, *LDL* low-density lipoprotein

## Discussion

The present study investigated the demographic and clinical characteristics of patients with T2DM in Greece and the level of their glycaemic control. It enrolled 1141 patients and used a dedicated web-based registry to collect data from five specialized diabetes centers operating within Greek hospitals.

57.1% of patients registered and assessed were not achieving the goal of HbA1c < 7% during the study period. This is in accordance with previous Greek and global studies reporting that even though treatment guidelines suggest a rapid progression in the therapeutic algorithm to achieve good glycaemic control [23], the percentage of patients achieving the target HbA1c score remains low [26–28]. In particular, in the PANORAMA study Greek results it was estimated that 33% of 375 patients recorded an HbA1c level above 7% in the index visit [27]. The percentage of patients not achieving glycaemic control in a recent national study was equal to 47% [35].

In the univariate analysis, poor glycaemic control was associated with the type of pharmacological treatment. Those receiving oral medication, or a combination of oral and injectable treatment were less likely to be controlled than those receiving injectable treatment only. In the multivariate analysis, results have been adjusted by duration of treatment. Consequently, this variable cannot be the single explanation of the observed association. A previous study reported that injectable treatment with or without oral medications was associated with worse glycaemic control compared to diet and exercise alone [36] Moreover, Liatis et al., [35] indicated that patients treated with insulin tend to have worse levels of glycaemic control due to insulin being prescribed in latest stages of the disease. A possible explanation may be that the effect of GLP-1RA based-therapies mediates the relationship between injectable treatments and glycaemic control found in our study. It is also interesting that previous studies have reported the complexity of pharmaceutical treatment as a predictor of poor glycaemic control, with patients receiving more than 5 medications recording worse glycaemic control [36, 37].

Poor glycaemic control was independently associated with a higher BMI. Patients with BMI higher than 25 had a 2-fold higher risk for poor glycaemic control. A previous study among insulin-receiving patients with T2DM has associated lower BMI with worse glycaemic control [38]. The variability of these results may be explained by the multifactorial pathogenetic pathways of T2DM resulting in a smaller significance of personal physical factors in the overall management of the disease.

Further, better glycaemic control was observed in patients with a better lipid profile. High HDL levels and low LDL levels were independently associated with an HbA1c level < 7 [39]. This can be explained by the nature of the disease, affecting multiple metabolic pathways. The inverse association between HDL-C and HbA1c may be due to the rise in TGs in poorly controlled patients, which in turn, are inversely correlated to HDL-C. Hence, low HDL-C in poorly controlled patients may be the consequence of hyperglycemia rather than a causative factor**. T**hese patients constitute a high-risk group that should be managed properly to reduce the risk of CV risk and mortality [40].

Contrary to past studies, there was no statistically significant association between duration of treatment and glycaemic control. The duration of treatment has been reported as a predictor of poor glycaemic control in a number of previous studies [24, 37–40]. Moreover, no association between poor glycaemic control and age was confirmed. However, past studies have reported an association of younger age in patients with T2DM with poor glycaemic control, especially in patients younger than 40 years of age [24, 37–41]. The lack of statistical significance in this association in the present study may be attributed to the relatively small percentage of patients in this age group.

This analysis was performed on the HbA1c level recorded during the first visit of the study period. In patients, in whom the last HbA1 measurement was recorded outside the study period, this information was missing. Therefore, these patients were excluded from the assessment of their glycaemic control.

High prevalence of risk factors for CVD were confirmed among the study population. 61.5% of study population suffered from dyslipidemia and 58.9% from hypertension. These findings are in line with the existing literature, which has reported an even higher prevalence of these conditions in the diabetic population in both national and international settings. More specifically, in the population of the Diabetes Collaborative Registry, the prevalence of hypertension was estimated at 76.3% and of dyslipidemia at 70.7% [30], whereas Akhter et al., [42] estimated hypertension and dyslipidemia rates at 55.5% και 41.3%, respectively in a total of 876 patients. Moreover, among the Greek subgroup of the PANORAMA study hypertension criteria were met by 30.2% of the sample [27].

A very high prevalence of other established comorbidities was also reported among study population. 21.6% was reported as having Coronary Artery Disease and 8.2% heart failure. Those results are consistent with other large epidemiological studies that report a high prevalence of heart disease among patients with T2DM. In a recent Greek study, the prevalence of self-reported heart disease among patients with T2DM was 24% [43]. Additionally, the presence of stroke or transient ischemic attach was reported among 4.6 and 4.4% of sample respectively. Patients in the group of already established cardiovascular disease are the primary target for interventions aiming to reduce overall CV risk. It is interesting to investigate the treatment approach in this category of patients and the adherence to newer treatment guidelines specifically targeted at this group [23].

On the other hand, known CV risk factors were very common among study population, with active smoking recorded in 31.7% and lack of physical activity in 85.3% of sample. Therefore, this study confirms the urgency for a multi-systematic approach regarding the management of all patients with T2DM to reduce high morbidity and mortality associated with each CV event [44].

This study included multiple centers and used a standardized web-based registry to collect data. This standardized approach to data collection reduces variability in data management among different centers and facilitates real-time data monitoring, during each patient visit, thus reducing the effect of recall bias.

This study was limited by the fact that laboratory results were recorded by different laboratories in each participating hospital. This may have resulted in high variability, especially in the measurement of specific parameters, such as HbA1c, where the method of measurement is critical. Another limitation is that patients enrolled in this study were treated in specialized diabetes centers operating in large hospitals. These patients are expected to be more challenging, with a higher prevalence of comorbidities and complications than those treated in smaller units or primary care facilities. Participating centers were solely specialized diabetes clinics operating in large urban areas. Therefore study findings should be generalized with caution in the national setting, as variations in patient characteristics and management may exist.

## Conclusions

Prevalence of risk factors for CVD and other comorbidities deemed high among the study population. Furthermore, a small proportion of the sample was engaged in healthy lifestyle behaviors. Inadequate lipid control, family history of diabetes and presence of obesity (ΒΜΙ ≥ 30 kg/m^2^) were associated with poor glycaemic control among study sample, whereas use of injectable antidiabetic agents was less likely to be associated with poor glycaemic control. These findings indicate how complex optimal management for patients with T2DM is, highlighting the need for tailored interventions in high-risk subpopulations with T2DM.

## Data Availability

The datasets used and analyzed during the current study are available from the corresponding author on reasonable request.

## Abbreviations

BMI: Body mass index
BP: Blood pressure
CKD: Chronic kidney disease
CVD: Cardiovascular disorders
DM: Diabetes mellitus
eGFR: Estimated glomerular filtration rate
HbA1c: Hemoglobin A1c
HDL: High-density lipoprotein
LDL: Low density lipoprotein
T2DM: Type 2 diabetes mellitus
TG: Triglycerides
TIA: Transient ischemic attack
WHO: World Health Organization
